# A Web-Based Cognitive Behavioral Therapy, Mindfulness Meditation, and Yoga Intervention for Posttraumatic Stress Disorder: Single-Arm Experimental Clinical Trial

**DOI:** 10.2196/26479

**Published:** 2022-02-28

**Authors:** Megan A Kirk, Bilal Taha, Kevin Dang, Hugh McCague, Dimitrios Hatzinakos, Joel Katz, Paul Ritvo

**Affiliations:** 1 School of Kinesiology and Health Sciences York University Toronto, ON Canada; 2 Yale Center for Emotional Intelligence Yale School of Medicine Yale University New Haven, CT United States; 3 Department of Electrical and Computer Engineering Faculty of Applied Science and Engineering University of Toronto Toronto, ON Canada; 4 Institute for Social Research York University Toronto, ON Canada; 5 Department of Psychology York University Toronto, ON Canada

**Keywords:** posttraumatic stress disorder, cognitive therapy, internet delivery, pupillometry, psychophysiology, PTSD, therapy, cognitive behavioral therapy, mindfulness, intervention

## Abstract

**Background:**

Posttraumatic stress disorder (PTSD) is a debilitating, undertreated condition. The web-based delivery of cognitive behavioral therapy supplemented with mindfulness meditation and yoga is a viable treatment that emphasizes self-directed daily practice.

**Objective:**

This study aims to examine the effectiveness of a web-based cognitive behavioral therapy, mindfulness, and yoga (CBT-MY) program designed for daily use.

**Methods:**

We conducted an 8-week, single-arm, experimental, registered clinical trial on adults reporting PTSD symptoms (n=22; aged 18-35 years). Each participant received web-based CBT-MY content and an hour of web-based counseling each week. Pre-post outcomes included self-reported PTSD symptom severity, depression, anxiety, chronic pain, and mindfulness. Pre-post psychophysiological outcomes included peak pupil dilation (PPD) and heart rate variability (HRV). HRV and PPD were also compared with cross-sectional data from a non-PTSD comparison group without a history of clinical mental health diagnoses and CBT-MY exposure (n=46).

**Results:**

Pre-post intention-to-treat analyses revealed substantial improvements in PTSD severity (*d*=1.60), depression (*d*=0.83), anxiety (*d*=0.99), and mindfulness (*d*=0.88). Linear multilevel mixed models demonstrated a significant pre-post reduction in PPD (*B=*−0.06; SE=0.01; *P*<.001; *d*=0.90) but no significant pre-post change in HRV (*P*=.87). Overall, participants spent an average of 11.53 (SD 22.76) min/day on self-directed mindfulness practice.

**Conclusions:**

Web-based CBT-MY was associated with clinically significant symptom reductions and significant PPD changes, suggesting healthier autonomic functioning. Future randomized controlled trials are needed to further examine the gains apparent in this single-arm study.

**Trial Registration:**

ClinicalTrials.gov NCT03684473; https://clinicaltrials.gov/ct2/show/NCT03684473

## Introduction

### Background

Posttraumatic stress disorder (PTSD) is a complex, disabling condition prevalent in 6.1% to 9.2% of the North American population [1-4]. PTSD is associated with distressing memories of traumatic events, persistent avoidance, negative mood, and hyperarousal [5]. Severe symptoms are often comorbid with depression and chronic pain [6-8].

The hyperarousal of PTSD reflects sympathetic nervous system dominance [9-13] in contrast to sympathetic–parasympathetic states typical of regulated autonomic functioning [14]. Pupillometry assesses pupil constriction and dilation changes that reflect autonomic nervous system (ANS) alterations relevant to ANS balance–imbalance [15-21]. Another physiological marker, heart rate variability (HRV), also reflects ANS status [22], with high HRV indicative of healthy states and low HRV signaling chronic disruption and pathology [23,24]. This study used both physiological markers to assess PTSD treatment efficacy.

### Therapeutic Treatment Approaches for PTSD

Cognitive behavioral therapy (CBT) for PTSD is a psychotherapeutic intervention aimed at challenging dysfunctional thoughts and beliefs about the self and the world, and addressing emotional and behavioral patterns that impair social and emotional function [25,26]. Although CBT is one of the best-validated treatments for PTSD [25-31], current data suggest that certain PTSD subgroups (eg, severe symptom severity) are treatment resistant to cognitive-focused therapies [32,33]. As PTSD is associated with dysregulated bottom-up processes (eg, ANS dysregulation), mindfulness meditation (MM), with its emphasis on body awareness, has been identified as a promising adjunct to treatment. Mindfulness is defined as the nonjudgmental, moment-to-moment awareness of thoughts, emotions, and bodily sensations [34-36]. The most empirically supported mindfulness-based therapies include mindfulness-based stress reduction [35,37], mindfulness-based cognitive therapy [38], acceptance and commitment therapy [39], and mindfulness-based CBT via the web [40]. Meta-analytic findings suggest that stand-alone mindfulness-based interventions have a moderate effect (Hedges *g*=−0.44) on PTSD symptoms, with longer intervention duration associated with greater PTSD symptom reduction [41].

MM combined with CBT involves reframing habitual cognitive distortions and dysfunctional thought patterns while learning to experience emotions and bodily sensations with nonjudgmental acceptance [38,42]. Interventions of this kind have received the most validation in populations with major depression and comorbid anxiety and are known to be better than usual care in reducing major depression relapse and anxiety symptoms [42]. MM combined with CBT has also shown promise in treating PTSD symptoms [43-45], although trauma-related thoughts or emotions that surface during meditation can trigger or retraumatize patients, disrupting treatment efficacy [46-48].

Yoga is increasingly being recognized as a complementary therapeutic modality for a wide variety of mental health conditions because of its ANS effects and stimulation of the limbic system [49]. Yoga-based integration of breath awareness with sequenced physical movements can promote autonomic regulation [46,47] and interoceptive awareness, which are especially relevant to survivors of PTSD who dissociate physically to cope with uncomfortable visceral sensations [50,51]. Interoceptive awareness is crucial for self-regulation and the integration of unprocessed aspects of trauma connected to bodily experience [51]. Therefore, yoga can further supplement cognitive therapy and MM to help restore self-regulation [47,51]. Although yoga, mindfulness, and their combined application as PTSD treatment have been investigated with proven efficacy [52], this is the first known study that has investigated the fuller combination of CBT, MM, and yoga for PTSD on both psychometric and psychophysiological outcomes.

Other promising CBT developments involve web-based applications that enable 24/7 accessibility [40]. Notably, meta-analytic findings provided evidence of web-based CBT as a clinically meaningful treatment for PTSD and comorbid depression and anxiety [53,54] with equal efficacy relative to in-person CBT [53]. Web-based CBT has the potential to be effective in the post-COVID-19 era, given the benefits of wide accessibility and scalability, and the possibility of reduced treatment costs.

This study examined the effectiveness of an 8-week intervention combining web-based CBT, mindfulness, and yoga (CBT-MY) for PTSD. Analyses assessed psychometric improvement and psychophysiological changes via pupillometric and HRV indicators of autonomic function. Our hypotheses were the following:

1. Hypothesis 1: Clinically significant reductions in PTSD Clinician-Administered Posttraumatic Stress Scale (CAPS-5) scores, defined as a minimum reduction of 10 points, were predicted at postintervention.
2. Hypothesis 2: Clinically significant improvements in comorbid mental health outcomes of depression, anxiety, pain, and mindfulness were predicted at postintervention.
3. Hypothesis 3: Reductions in peak pupil dilation (PPD) and increases in HRV were predicted at postintervention.

## Methods

### Study Design

This study was a registered, single-arm, experimental clinical trial (ClinicalTrials.gov NCT03684473) approved by the York University human participants research and ethics committee.

### Population

Participants (aged 18-35 years) were full-time students enrolled at York University, Toronto, Canada, and recruited through classroom announcements, flyers, and an undergraduate research pool. Eligibility criteria included exposure to trauma and a clinical diagnosis of threshold PTSD symptoms measured by the CAPS-5 or threshold symptoms on the PTSD Checklist for the Diagnostic and Statistical Manual of Mental Disorders, fifth edition (DSM-5) civilian version. Exclusion criteria were (1) current trauma exposure within the past 30 days, (2) significant suicide risk, (3) substance abuse, and (4) active psychological treatment. A cross-sectional convenience sample of students (aged 18-35 years) with no prior history of diagnosed mental health disorders or treatment were recruited for PPD and HRV autonomic function comparisons, which were collected during a modified 25-minute validated computer-based protocol of rest, stress, and guided meditation (GM) [55,56].

### Sample Size

A power calculation was conducted based on hypothesis 1 to detect a minimal clinically important difference in PTSD symptom severity, defined as a CAPS-5 symptom reduction of ≥10.4 points or a z score change of ≥0.8 SDs [57]. On the basis of Cohen [58] large effect size (*d*=0.80), power of 0.8, and α<.05 (correlation among factors, *r*=0.50), it was estimated that a minimum sample of N=15 would provide ample testing power [58,59]. We anticipated 20% attrition (n*=*3), and planned to over enroll (N=18) to detect pre-post, within-group differences for hypothesis 1 [59,60].

### Procedure

Interested participants emailed the study coordinator and were sent a prescreen survey (eg, trauma exposure, current symptoms, and suicidal ideation). Participants who met the inclusion criteria attended a 90-minute in-person meeting to further assess symptoms and provide written informed consent. Participants were screened for trauma exposure using the Life Events Checklist for DSM-5 to establish the DSM-5 Criterion A and underwent the CAPS-5 interview with a trained clinician. Participants suspended current psychological but not psychiatric treatment during the 8-week intervention period. Within 1 week of the CAPS-5 interview, participants completed demographic and psychometric questionnaires and laboratory assessment of psychophysiological variables (time point 1 [T1]; baseline [BL] measurements).

### Intervention

The 8-week CBT-MY intervention was initiated after the laboratory assessment, where each participant received password-protected web-based program access. The program comprised eight CBT-themed modules (eg, cognitive distortions, negative self-talk) containing (1) 56 unique daily MM exercises focused on breath awareness, progressive relaxation, and nonjudgmental body awareness; (2) eight 20-minute trauma-informed yoga videos [61]; and (3) 10 mindfulness-of-breath practices (Multimedia Appendix 1). Participants had access to the prerecorded CBT-MY content 24 hours a day between the counseling sessions. Participants met weekly for a 60-minute web-based session with a CBT counselor, who attended weekly supervision sessions with a clinical psychologist (PR). Participants were instructed to engage in 90 minutes of self-directed CBT-MY practice per week. All psychological and psychophysiological variables were reassessed at postintervention (time point 2 [T2]; postintervention measurements).

### Primary Outcome Measures

#### Life Events Checklist for DSM-5

The Life Events Checklist for DSM-5 was used to establish trauma exposure to 17 stressful events associated with PTSD [62]. Respondents self-reported exposures based on 6 responses: (1) *happened to me*, (2) *witnessed it*, (3) *learned about it*, (4) *part of job*, (5) *not sure,* (6) *doesn’t apply*.

#### CAPS-5 Interview

PTSD symptom severity was assessed using the CAPS-5, a 30-item structured in-person interview that confirmed the current (ie, past month) diagnosis of PTSD [63]. All CAPS-5 interview notes and scoring were independently assessed by a second clinician with no study relationship. Discrepancies were resolved through consultation with a supervising clinical psychologist. The CAPS-5 total severity score had a high internal consistency (α=.88) with strong interrater reliability (intraclass correlation coefficient=0.91) and good test–retest reliability (intraclass correlation coefficient=0.78 [63]).

#### PTSD Checklist for the DSM-5 Measure

The PTSD Checklist for DSM-5 (PCL-5), a 20-item self-report measure of DSM-5 PTSD symptoms, was used as an additional measure of PTSD symptom severity [64]. The PCL-5 has demonstrated good internal consistency (α=.94) and test–retest reliability (*r=*0.66-0.82 [64,65]).

### Secondary Outcome Measures

#### Beck Depression Inventory-2

The Beck Depression Inventory-2 (BDI-2 [66]), a 21-item self-report survey, was used to assess depression symptom severity and has demonstrated internal consistency [67] and test–retest reliability [68-70].

#### Beck Anxiety Inventory

The Beck Anxiety Inventory (BAI [71,72]), a 21-item self-report inventory, was used to assess symptoms of anxiety and has demonstrated internal consistency and test–retest reliability [71-74].

#### Pain Catastrophizing Scale

The Pain Catastrophizing Scale (PCS [75]) is a self-report measure of pain catastrophizing through pain-related thoughts, perceived helplessness, and exaggerated attentional focus on the threat of pain stimuli [75]. The PCS comprises rumination (α=.87), magnification (α=.60), and helplessness (α=.79) subscales [75].

#### Brief Pain Inventory

The Brief Pain Inventory (BPI [76,77]) is a 16-item, self-report questionnaire that measures the severity of *worst* and *least* pain in the past 24 hours, as well as participants’ *average* and present pain (*right now*). It has demonstrated reliability and validity in patients with chronic nonmalignant pain [78].

#### Five Facet Mindfulness Questionnaire

The Five Facet Mindfulness Questionnaire (FFMQ [79]) is a valid 39-item survey that measures five mindfulness subscales: (1) *observing*, (2) *describing*, (3) *acting with awareness*, (4) *nonjudging of experience*, and (5) *nonreactivity to inner experience*. The subscales have demonstrated good construct validity and internal consistency, with α values ranging from .75 to .91 [80-82].

Demographic characteristics and intervention adherence variables were assessed via an investigator-initiated questionnaire.

### Objective Outcome Measures

#### Autonomic Function

Autonomic function was assessed via PPD and HRV using a modified version of a validated 25-minute computer protocol [55,56]. A 5-minute BL rest phase was followed by a 10-minute artificial stress task (stress phase) and a 10-minute GM (recovery phase). During BL and GM, participants viewed a moving fixation cross on a computer screen that randomly changed locations in a 9-square grid every 10 seconds. The modified stress task (emotional stress task) comprised 60 International Affective Picture System (IAPS [83]) images depicting fear, sadness, anger, or frustration to evoke stress responses (Multimedia Appendix 2). Images were presented one at a time for 10 seconds [17,18]. The IAPS provides normative ratings of affect for images based on the Self-Assessment Manikin 9-point rating scale: 9 represents a high rating (ie, high pleasure and high arousal), 5 indicates a neutral rating, and 1 represents a low rating (ie, low pleasure and low arousal [84]). All images were carefully screened for acceptability and approved by a clinical psychologist who supervised the trial. The 60 selected images had a moderately low pleasure rating (mean 3.05, SD 1.63, range 1.79-4.31) and a moderately high arousal rating (mean 5.52, SD 2.13, range 3.93-6.96) [84]. The protocol was conducted in a standardized dimly lit, sound-attenuated room. A chin rest at 65 cm from the computer screen was used to optimize the pupillometric measurement.

#### Pupillometry

The PPD response was used to assess autonomic balance in conjunction with high-frequency HRV (HF-HRV) using the TobiiPro Glasses 2.0 eye tracking device [85]. PPD was assessed while participants viewed negative images on a computer screen. The data were cleaned using the recommendations of Siegle et al [86]. Trials with >50% blinks resulted in exclusion (1/18, 6%). Changes in PPD were recorded every 20 milliseconds (60 Hz) for 10 seconds following the onset of an image stimulus using Tobii Pro Software [85]. The BL pupil diameter for both the left and right pupils was calculated as the mean pupil size during the 200 milliseconds preceding the onset of the fixation crosses at BL. The resting pupil diameter was calculated as the weighted sum of both pupils. Autonomic PPD was calculated as the maximum pupil response in the first 500 milliseconds after image onset and averaged across 60 images. The first 300 to 500 milliseconds of measurement is recommended for pupillary response to stimuli where no saccades (eg, ballistic fast movements of the eyes and image avoidance) could be initiated [87,88]. Data were inspected for noise and outlier samples using Python software before analysis. Artifacts were identified and removed based on the guidelines of Kret and Sjak-Shie [89]. After the eyeblinks and artifacts were removed, a linear interpolation function was applied to the data to accommodate for missing values. To increase the temporal resolution and smoothness of the data, the data points were resampled with interpolation to a high sampling rate (1000 Hz) based on recommendations [89]. The resulting time-series signal was then smoothed using a zero-phase low-pass filter with a cutoff frequency of 4 Hz [90]. The final step was to calculate the relative pupil size change (percentage change) from the calculated resting pupil size based on recommendations by the Tobii Pro Studio software [81,91]. The Tobii Pro 2.0 glasses have optical sensors that calculate the PPD measure based on eye model algorithms. As the glasses do not report the actual physical pupil diameter, relative change measures such as percentage change in dilation were used to indicate physiological arousal [81,91].

#### HF-HRV Measure

HF-HRV is an established measure of parasympathetic control [92] and was acquired using a 3-lead electrocardiography (ECG) machine. ECG signals were digitized using a PowerLab 4/35 acquisition system (ADInstruments). LabChart 8 software was used to record and analyze the digitized signals at a sampling rate of 1000 Hz. ECG data were collected and prepared in accordance with the standards set by the 1996 Task Force of the European Society of Cardiology and the North American Society of Pacing and Electrophysiology [92]. LabChart 8 uses automated ECG interpretation, which indicates ectopic and normal beats. ECG recordings were visually inspected to ensure clear PQRST heart beat wave formations free of artifacts, arrhythmias, and ectopic beats and were manually adjusted if beat markers were missing or misplaced. HF-HRV (0.15-0.40 Hz) is conventionally an estimate of short-term (5-minute) recordings of vagal modulation and parasympathetic nervous system activity in HRV [92] and was the primary outcome variable measured in this analysis. Respiration was collected using a respiratory belt and was controlled for in the analyses. The average cyclical rate was calculated using LabChart 8.

#### Program Adherence and Satisfaction

The attendance rate and duration of counseling calls were tracked, as was the self-reported 7-day recall of time spent in self-directed practice. Google Analytics was linked to the intervention website to monitor the web-based website analytics of visitors during the treatment period. Program usefulness and satisfaction were assessed at postintervention.

### Statistical Analysis

Analyses were performed using SPSS Statistics version 23.0 (IBM Corporation).

#### Hypotheses 1 and 2

The exposure variable was time (BL and postintervention, ie, T1 and T2). The outcomes were PTSD severity, PTSD comorbidities, mindfulness, HF-HRV, and PPD. All outcome variables were treated as continuous.

#### Hypotheses 1 and 2 Testing: Improvement in Psychometric Outcomes Individually

Paired-sample *t* tests (2-tailed; comparing T1 and T2) were conducted to assess changes in psychometric outcomes using intention-to-treat (ITT) analysis (last observation carried forward) and per-protocol (PP) analysis. Cohen *d* was used to evaluate effect size. Box plots were used to check for outliers. Extreme outliers were those located at >3 box lengths from the upper and lower quartiles. The Shapiro-Wilk test was used to assess the normality of the difference scores.

#### Hypothesis 3: Pre-Post Improvement in Psychophysiological variables of HF-HRV and PPD

Before the analyses, raw HF-HRV and PPD data were examined for normality. HF-HRV was natural log transformed to adjust for positively skewed data (Shapiro-Wilk test; *P*<.001). The study did not have a control group that completed pre-post measurements; instead, comparisons were undertaken with a convenience sample of BL-only participants without PTSD who did not receive the intervention.

Linear mixed effects (LME) models using multilevel regression allows for the inclusion of all cases in unbalanced data [93]. Thus, the non-PTSD convenience sample that completed only the initial BL time point could be entered into the model [93]. Entering all data into one model represents a more parsimonious and accurate analytic approach [93]. A 2-level LME was conducted with the HF-HRV (natural log transformed) or PPD set as the outcome variable. The LME had two levels; level 1 included the individual measurements on a given participant at a given time (eg, only BL for some participants), and level 2 was the participant. The fixed factors were coded as follows: treatment group (0=participants with PTSD who received intervention and 1=convenience, BL-only participants without PTSD), time (0=BL vs 1=postintervention), and protocol condition (eg, 0=BL rest 0-5 minutes, 1=emotional image stress task [EST] 0-5 minutes, 2=EST 5-10 minutes, 3=GM 0-5 minutes, and 4=GM 5-10 minutes). For the within-person repeated measures, participants with PTSD had two repeated measures: time (BL vs postintervention) and protocol condition (BL, EST 0-5 minutes, EST 5-10 minutes, GM 0-5 minutes, and GM 5-10 minutes). The participants without PTSD had one repeated measure: protocol condition (BL, EST 0-5 minutes, EST 5-10 minutes, GM 0-5 minutes, and GM 5-10 minutes). Participants without PTSD were coded for time as 0=BL and for treatment group as 0=did not receive intervention. The model included a subject intercept slope to account for random effects. Significant main effects of treatment group, time, and protocol conditions were evaluated with pairwise comparisons using a Bonferroni type 1 error rate correction. Post hoc analyses using independent and paired-sample *t* tests (2-tailed) were conducted to ascertain where significant differences emerged. Objective outcome measures were conducted using PP analysis to determine the treatment effect on the psychophysiology of participants who complied with the protocol. Exploratory bivariate correlations were examined between psychometric and psychophysiological data to analyze possible associations.

## Results

### Overview

**Figure 1:**
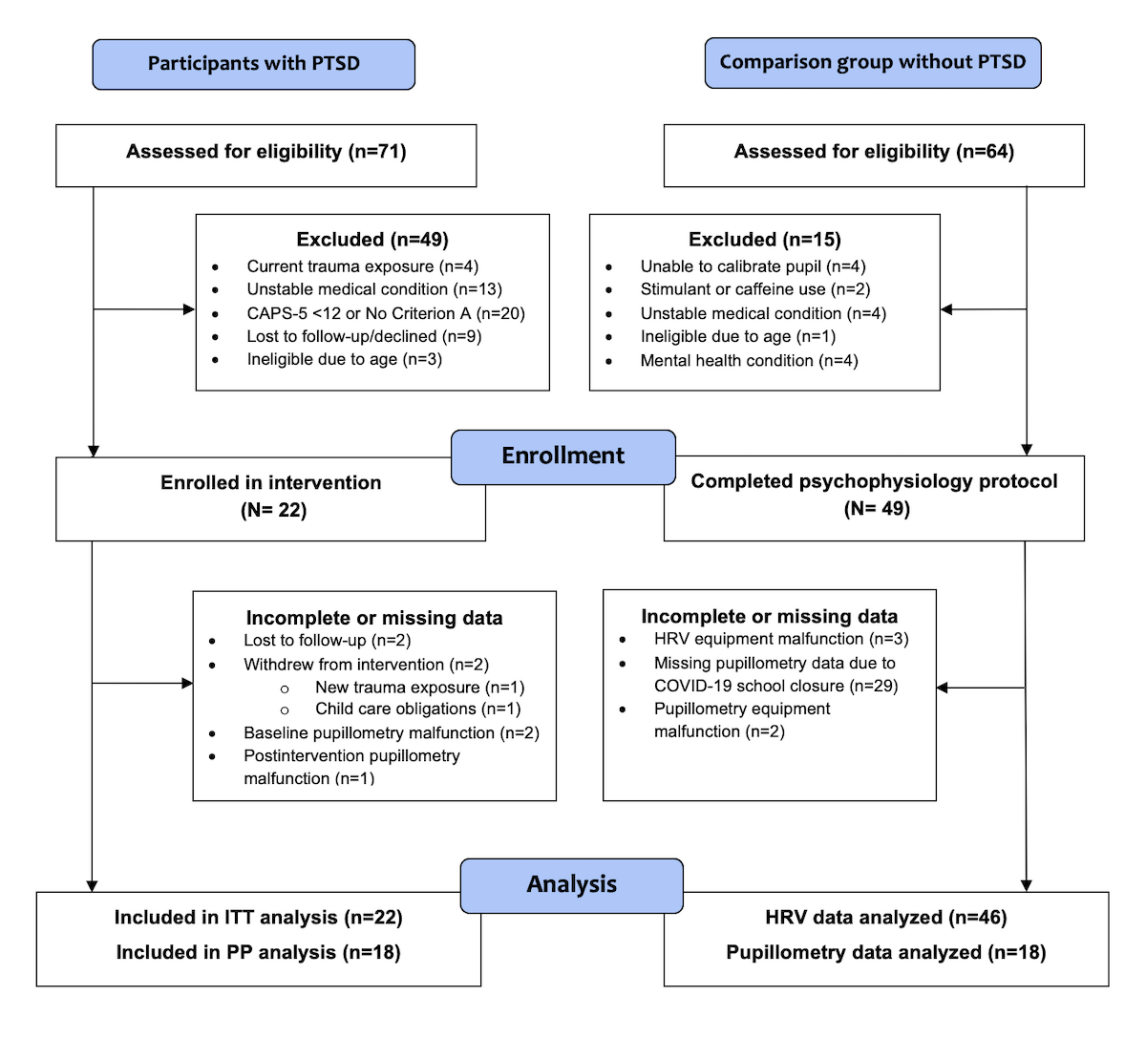
CONSORT (Consolidated Standards of Reporting Trials) flow diagram of participant enrollment, follow-up, and analysis. CAPS-5: Clinician-Administered Posttraumatic Stress Scale; HRV: heart rate variability; ITT: intention-to-treat; PP: per-protocol; PTSD: posttraumatic stress disorder. Unstable medical condition (ie.current suicide ideation, substance abuse, current medical treatment).

Overall, 71 adults aged 18 to 35 years were screened for eligibility from October 2018 to December 2019 (Figure 1). Of the 71 adults, 49 (69%) were excluded. The reasons for exclusion were current exposure to trauma (4/49, 8%), high suicide risk or an unstable medical condition (14/49, 29%), a CAPS-5 score <12 or no clear trauma exposure (Criterion A; 20/49, 41%), unable to be contacted (9/49, 18%), or ineligible age (3/49, 6%). On the basis of the inclusion criteria, of the 71 adults, 22 (31%) adults with PTSD provided consent and were enrolled in the study. Of the 22 participants, 2 (9%; 1/2, 50% in week 2 and 1/2, 50% in week 7) withdrew from the study, and an additional 2 (9%; 1/2, 50% in week 2 and 1/2, 50% in week 8) were lost to follow-up. We compared the HF-HRV data of participants with PTSD with the HF-HRV data of 46 participants without PTSD and pilot pupillometry data findings for 18 participants without PTSD.

### Demographics of Participants With PTSD

The demographic characteristics of the participants with PTSD are presented in Table 1. Participants were aged 26.4 (SD 4.45) years on average, and most were female (18/22, 82%). Most reported a clinical mental health diagnosis confirmed by a clinical mental health professional (18/22, 82%). Of the 22 participants, 14 (64%) disclosed ≥2 diagnoses, and 11 (50%) reported currently taking prescription medication for a mental health condition.

Table 2 presents a summary of the clinical mental health and trauma exposure variables. Anxiety (14/22, 64%), depression (13/22, 59%), and PTSD (12/22, 55%) were the most common diagnoses. Participants reported an average of 3.5 (SD 2.89) years since the last trauma exposure, with a mean of 11.5 (SD 5.92) lifetime trauma exposures. Most participants experienced direct trauma (mean 5.14, SD 1.86), with physical assault being the most common trauma (19/22, 86%), followed by sexual assault (17/22, 77%). Most participants experienced direct childhood trauma exposure (19/22, 86%) that, on average, occurred as early as the age of 6.66 (SD 4.40) years and persisted for 9.97 (SD 4.62) years*.* Most childhood abuses were perpetrated by a parent (14/22, 64%), multiple family members (6/22, 27%), or a single family member (4/22, 18%).

**Table 1:** Demographic characteristics of posttraumatic stress disorder intervention participants (N=22).

Variables			Values
Age (years), mean (SD)			26.4 (4.45)
**Gender, n (%)**			
	Female	18 (82)	
	Male	3 (14)	
	Nonbinary	1 (5)	
**Ethnicity, n (%)**			
	White (North American or European)	13 (59)	
	Black (African American or Caribbean)	1 (5)	
	Middle Eastern (Persian or Arabian)	3 (14)	
	Asian (Chinese)	1 (5)	
	South Asian (Pakistani or Indian)	1 (5)	
	Mixed race	3 (14)	
**Immigration status, n (%)**			
	Landed immigrant	10 (46)	
	Canadian citizen	12 (55)	
**Education status, n (%)**			
	Undergraduate degree in progress (BA^a^ and BSc^b^)	16 (73)	
	Second degree in progress (MA^c^, PhD, and second bachelor’s degree)	6 (27)	
**Marital status, n (%)**			
	Single	12 (55)	
	Dating	6 (27)	
	Common law or married	4 (17)	
**Employment status, n (%)**			
	Unemployed	5 (23)	
	Part time (0-35 hours/week)	15 (68)	
	Full time (≥35 hours/week)	2 (9)	
**Pre-existing physical health condition, n (%)**			
	None	11 (50)	
	Obesity or overweight	4 (17)	
	Fibromyalgia or chronic fatigue syndrome	2 (11)	
	Autoimmune disorder	2 (11)	
	Other (ie, Tinnitus, IBS^d^, and PCOS^e^)	5 (28)	
	Prediabetes	1 (5)	
	≥2 conditions	4 (22)	
**Mental health diagnosis**			
	Clinical diagnosis, n (%)	18 (82)	
	≥2 diagnoses, n (%)	14 (64)	
	Total diagnoses, mean (SD)	2.27 (1.75)	
**Prescription medications for mental health**			
	None, n (%)	11 (50)	
	1 medical prescription, n (%)	6 (27)	
	≥2 medical prescriptions, n (%)	5 (23)	
	Total medications, mean (SD)	1.41 (2.02)	
**Mindfulness practice (min/day), n (%)**			
	0	16 (73)	
	<10	6 (27)	
	>10	0 (0)	

^a^BA: bachelor of arts.

^b^BSc: bachelor of science.

^c^MA: master of arts.

^d^IBS: irritable bowel syndrome.

^e^PCOS: polycystic ovary syndrome.

**Table 2:** Clinical mental health and trauma exposure variables of participants with posttraumatic stress disorder (PTSD; N=22).

Mental health and trauma exposure variables				Values
**DSM-5^a^ clinical mental health diagnosis, n (%)**				
	PTSD			12 (55)
	Depression			13 (59)
	Anxiety			14 (64)
	Borderline personality disorder			3 (14)
	Addiction or self-harm			3 (14)
	Multiple diagnoses (≥2 diagnoses)			14 (64)
	Other (ie, attachment disorder)			1 (5)
**Trauma exposure (LEC-5)^b^, mean (SD)**				
	*Happened to me* (directly)			5.14 (1.86)
	*Witnessed it*			2.27 (1.96)
	*Learned about it*			3.55 (3.61)
	*Part of job*			0.55 (2.35)
	Total trauma exposures			11.50 (5.92)
	Time since last trauma exposure (years)			3.50 (2.89)
**Direct trauma exposure category, n (%)**				
	Physical assault			19 (86)
	Sexual assault (eg, rape or attempted rape)			17 (77)
	Unwanted sexual experience (eg, groping or sexual harassment)			17 (77)
	Transportation accident			10 (46)
	Repeated trauma exposure^c^			20 (91)
	Other (eg, psychological abuse)			18 (82)
**Childhood trauma exposure**				
	*Happened to me*, n (%)			19 (86)
	Age of first memory (years), mean (SD)			6.66 (4.40)
	Duration of childhood trauma (years), mean (SD)			9.97 (4.62)
	**Childhood abuser, n** **(** **%)**			
		Parent	14 (74)	
		Family member	4 (21)	
		Multiple family members	6 (27)	
		Friend or acquaintance	2 (11)	
		Stranger	1 (5)	
	**Type of childhood trauma, n (%)**			
		Physical abuse only	9 (47)	
		Sexual assault	9 (47)	
		Verbal emotional or psychological abuse	16 (84)	
**Past mental health treatment, n (%)**				
	Pharmacotherapy			11 (50)
	Talk therapy			20 (91)
	EMDR^d^			4 (18)
	Hospitalization			3 (14)
	Alternative or complementary medicine			4 (18)
**Family history of mental health, n (%)**				
	Yes			15 (68)
	No			7 (32)

^a^DSM-5: Diagnostic and Statistical Manual of Mental Disorders, fifth edition.

^b^LEC-5: Life Events Checklist for Diagnostic and Statistical Manual of Mental Disorders, fifth edition.

^c^Repeated by the same perpetrator or abuser or repeated type of trauma exposure.

^d^EMDR: eye movement desensitization and reprocessing.

### Treatment Response on Psychometric Outcomes

#### Hypothesis 1

Table 3 shows the mean and SD for each psychometric measure at T1 and T2, as well as the mean percentage change from T1 to T2 for ITT and PP approaches. Pre-post analysis revealed a significant reduction in CAPS-5 PTSD symptom severity from BL to postintervention for both ITT (Δmean=−19.18, SD 12.38; *t*_21_=7.27; *P*<.001) and PP (Δmean=−23.44, SD 9.14; *t*_17_=10.88; *P*<.001). Cohen *d* was calculated as 1.42 (ITT), which equates to a large effect size [58]. The largest symptom severity reduction was in Criterion D: negative cognition and mood symptoms (Δmean=−7.59, SD 5.23; *t*_21_=6.81; *P*<.001; *d*=1.39). Overall, the ITT results indicated that the 8-week intervention reduced PTSD symptom severity (Table 3) by 37.9%. This approximately 2-fold reduction in symptom severity surpassed the hypothesized minimal clinically important difference of a 10-point reduction.

**Table 3:** Pre- (time point 1 [T1]; baseline measures) and postintervention (time point 2 [T2]) psychometric outcomes for intention-to-treat (ITT; N=22) and per-protocol (PP; N=18) analyses*a*.

Psychometric measure		ITT (N=22)					PP (N=18)			
		T1, mean (SD)	T2, mean (SD)	Δ score (%)	Cohen *d*	T1, mean (SD)		T2, mean (SD)	Δ score (%)	Cohen *d*
**PTSD^b^ (CAPS-5^c^)**										
	Criterion B (intrusion)	12.23 (2.86)	*7.77* (*3.61*)^d^	–36.5	*1.37*	12.39 (2.39)		*6.94* (*3.23*)	–44.0	*1.92*
	Criterion C (avoidance)	5.18 (1.89)	*2.86* (*2.21*)	–44.8	*1.13*	5.06 (2.01)		*2.22* (*1.83*)	–56.1	*1.48*
	Criterion D (cognition or mood)	17.41 (4.62)	*9.82* (*6.95*)	–43.6	*1.29*	16.50 (4.50)		*7.22* (*4.39*)	–56.2	*2.09*
	Criterion E (arousal or reactivity)	12.27 (4.32)	*7.73* (*4.63*)	–37.0	*1.01*	12.22 (4.49)		*6.67* (*4.13*)	–45.4	*1.29*
	Total symptom severity	47.09 (9.09)	*27* (*15.22*)	–42.7	*1.60*	46.17 (9.56)		*21.61* (*10.54*)	–53.2	*2.44*
	Total symptoms (out of 20)	15.68 (2.10)	*9.73* (*5.32*)	−37.9	*1.47*	15.33 (2.14)		*8.11* (*4.40*)	–47.1	*2.09*
PTSD (PCL-5^e^)		48.14 (11.94)	*28.95* (*17.67*)	–39.9	*1.27*	45.56 (11.35)		*22.11* (*10.23*)	–51.5	*2.01*
Depression (BDI-2^f^)		28.14 (13.70)	*16.27* (*15.02*)	–42.2	*0.83*	25.78 (13.39)		*11.28* (*10.65*)	–56.2	*1.34*
Anxiety (BAI^g^)		29.68 (9.54)	*19.36* (*11.32*)	–34.8	*0.99*	28.33 (8.88)		*15.72* (*7.61*)	–44.5	*1.60*
Pain catastrophizing (PCS^h,i^)		20.00 (13.73)	*10.85* (*8.59*)	–45.6	*0.80*	22.00 (13.59)		*11.18* (*8.66*)	−49.2	*0.82*
Pain severity (BPI^j^)^i^		4.10 (1.01)	*2.48* (*1.68*)	–39.5	*1.17*	4.10 (1.01)		*2.48* (*1.68*)	–39.5	*1.17*
Pain interference (BPI)^i^		3.29 (1.39)	*2.10* (*1.74*)	–36.2	*0.75*	3.29 (1.39)		*2.10* (*1.74*)	–36.2	*0.75*
**Five-facet mindfulness (FFMQ^k^)**										
	FFMQ observing	25.09 (8.31)	27.45 (5.86)	9.4	—^l^	24.61 (8.79)		27.50 (5.98)	11.7	—
	FFMQ describing	25.73 (7.16)	27.49 (6.54)	6.8	—	25.75 (7.30)		28.06 (6.45)	9.0	—
	FFMQ awareness	20.95 (6.89)	*25.18* (*6.37*)	20.2	*0.64*	21.33 (6.86)		*26.50* (*5.42*)	24.2	*0.84*
	FFMQ nonjudging	20.55 (5.16)	*26.41* (*6.95*)	28.5	*0.96*	20.33 (5.57)		*27.50* (*7.16*)	35.3	*1.12*
	FFMQ nonreactivity	15.87 (5.17)	*20.55* (*6.05*)	29.6	*0.83*	16.56 (5.11)		*22.28* (*4.87*)	34.5	*1.15*
	FFMQ total score	108.18 (19.12)	*127.18* (*23.84*)	17.6	*0.88*	108.61 (20.32)		*131.83* (*23.20*)	21.4	*1.06*

^a^Cohen *d* values are reported in absolute values, and intention-to-treat using the last observation was carried forward.

^b^PTSD: posttraumatic stress disorder.

^c^CAPS-5: Clinician-Administered Posttraumatic Stress Scale.

^d^Italicized values indicate significant differences.

^e^PCL-5: Posttraumatic Stress Disorder Checklist for the Diagnostic and Statistical Manual of Mental Disorders, fifth edition.

^f^BDI-2: Beck Depression Inventory-2.

^g^BAI: Beck Anxiety Inventory.

^h^PCS: Pain Catastrophizing Scale.

^i^n=13 because of the addition of scale after 6 months from recruitment.

^j^BPI: Brief Pain Inventory.

^k^FFMQ: Five Facet Mindfulness Questionnaire (in this subscale, higher values indicate improvement).

^l^Nonsignificant values are not reported.

#### Hypothesis 2

There were significant reductions in symptom severity for PCL-5 (*t*_21_=6.44; *P*<.001), BDI-2 (*t*_21_=5.59; *P*<.001), BAI (*t*_21_=4.94; *P*<.001), PCS (*t*_12_=3.22; *P*=.01), BPI severity (*t*_6_=2.71; *P*=.04) and BPI interference (*t*_6_=3.20; *P*=.02), and FFMQ (*t*_21_=−5.00; *P*<.001). As shown in Table 3, effect sizes (Cohen *d*) of significant improvements ranged from 0.75 to 1.27, indicating a large effect, with PCL-5 exhibiting the largest reductions (*d*=1.27), followed by BAI (*d*=0.99), FFMQ (*d*=0.88), and BDI-2 (*d*=0.83). All secondary psychometric data demonstrated 17.5% to 45.6% symptom reduction supporting hypothesis 2.

### Treatment Effect on Psychophysiology Outcomes

#### HRV Analysis

**Figure 2:**
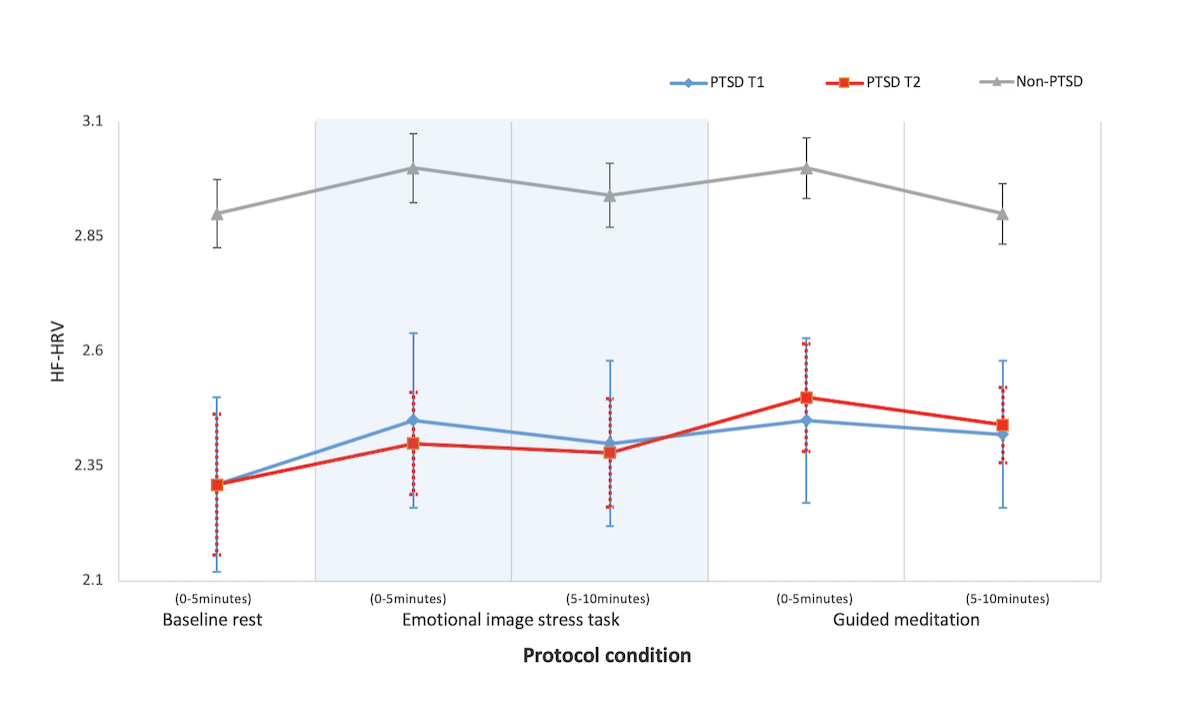
The HF-HRV of participants with PTSD at baseline (T1) and postintervention (T2) compared with participants without PTSD during the 25-minute protocol conditions of rest, stress, and guided meditation. HF-HRV is presented as a log10-transformed plot with SE bars. HF-HRV: high-frequency heart rate variability; PTSD: posttraumatic stress disorder; T1: time point 1; T2: time point 2.

Tables 4 and 5 present the descriptive statistics of ECG variables and HF-HRV among the participants with PTSD (n=18) and those without PTSD (n=46). Figure 2 depicts the HF-HRV across the 25-minute laboratory protocol. Participants without PTSD were, on average, significantly younger than the participants with PTSD (*d=*1.41; *P*<.001). Resting heart rate (HR) was significantly lower in participants without PTSD (mean 76.45, SD 11.09) than in participants with PTSD at BL (mean 86.43, SD 13.98; *d*=0.88; *P*=.002) and postintervention (mean 84.95, SD 10.76; *d*=0.71; *P*=.02).

Multilevel LME modeling on HF-HRV was performed with all 64 participants (eg, 18/64, 28% participants with PTSD and 46/64, 72% participants without PTSD) and revealed significant differences in HF-HRV (Table 6). The results revealed a significant main effect of treatment (eg, participants with PTSD vs participants without PTSD) group (*F*_1,65_=14.54; *P*<.001; ƞ^2^=0.10) and protocol (eg, BL, EST, GM) phase condition (*F*_4, 240_=6.33; *P*<.001; ƞ^2^=0.18) on HF-HRV. No significant main effect of time was found between BL and postintervention among participants with PTSD (*P*=.87), indicating no significant intervention effect on HF-HRV among participants with PTSD.

For the main effect of the treatment group, pairwise comparisons using Bonferroni type 1 correction revealed that the participants without PTSD had a significantly higher overall mean HF-HRV than participants with PTSD (B=0.55, SE 0.14; *t*_65.16_=3.81; *P*<.001) at *both* BL and postintervention. Furthermore, the differences in HF-HRV between participants with PTSD and participants without PTSD remained significant (*F*_4, 238_=7.70; *P*<.001; ƞ^2^=0.11) after controlling for respiration, indicating that the differences found were not because of the influence of respiration rate. Post hoc analyses for the main effect of protocol condition using BL rest phase as the reference indicated that HF-HRV was significantly higher during the first 5 minutes of the EST phase compared with BL rest phase for participants without PTSD (Δmean=0.104, SE 0.03; *d*=0.20; *P=*.002) and participants with PTSD at T1 (Δmean=0.14, SE 0.04; *d*=0.20; *P=*.002) but not for participants with PTSD at T2 (*P=*.26). Additionally, HF-HRV was significantly higher during the first 5 minutes of GM compared to BL rest phase for participants without PTSD (Δmean=0.09, SE 0.04; *d*=0.26; *P*=.02) and participants with PTSD at T1 (Δmean=0.14, SE 0.05; *d*=0.32; *P=*.09) and at T2 (Δmean=0.19, SE 0.07; *d*=0.30; *P*=.02).

**Table 4:** Estimated marginal means and log-transformed high-frequency heart rate variability comparisons among participants with posttraumatic stress disorder (PTSD; N=18) and a non-PTSD convenience sample cohort (N=46).

Variable	Participants with PTSD, mean (SD)		Participants without PTSD, mean (SD)
	T1^a^	T2^b^	T1
Age (years)	26.39 (4.23)	26.39 (4.23)	20.78 (3.64)^c,d^
BL^e^ heart rate (bpm^f^)	86.43 (13.98)	84.95 (11.76)	76.45 (11.09)^c,d^
BL respiration	16.32 (4.09)	16.30 (4.48)	16.93 (2.72)
BL RMSSD^g^	30.89 (26.02)	30.14 (19.54)	49.04 (25.69)
BL LF:HF^h^	1.84 (1.30)	2.87 (4.08)	1.43 (2.01)

^a^T1: time point 1 (baseline).

^b^T2: time point 2 (postintervention).

^c^Significant difference (*P*<.05) between participants without posttraumatic stress disorder and participants with posttraumatic stress disorder at time point 1 (baseline).

^d^Significant difference (*P*<.05) between participants without posttraumatic stress disorder and participants with posttraumatic stress disorder at time point 2 (postintervention).

^e^BL: baseline.

^f^bpm: beats per minute.

^g^RMSSD: root mean square of successive differences (between normal heartbeats).

^h^LF:HF: low-frequency to high-frequency heart rate variability ratio.

**Table 5:** Estimated marginal means (EMMs) and log-transformed high-frequency heart rate variability comparisons among participants with posttraumatic stress disorder (PTSD; N=18) and a non-PTSD convenience sample cohort (N=46) for the 25-minute protocol condition.

Variable	Participants with PTSD					Participants without PTSD	
	T1^a^		T2^b^		T1		
	EMM (SE)	Log_10_ (SE)	EMM (SE)	Log_10_ (SE)	EMM (SE)		Log_10_ (SE)
Baseline rest phase (5 minutes)	708.05 (224.96)	2.31 (0.19)	559.79 (147.12)	2.31 (0.18)	1467.45 (243.20)		2.90 (0.08)
EST^c^ 1 (0-5 minutes)	995.50 (344.73)	2.45 (0.19)	490.12 (131.23)	2.40 (0.13)	1827.16 (310.67)		3.00 (0.08)
EST 2 (5-10 minutes)	810.08 (264.90)	2.40 (0.18)	460.78 (103.26)	2.38 (0.14)	1446.24 (232.66)		2.94 (0.07)
GM^d^ 1 (0-5 minutes)	774.59 (207.90)	2.45 (0.18)	810.19 (357.26)	2.50 (0.14)	1547.99 (217.75)		3.00 (0.07)
GM 2 (5-10 minutes)	580.94 (150.65)	2.42 (0.16)	408.61 (87.78)	2.44 (0.10)	1220.34 (175.18)		2.90 (0.07)

^a^T1: time point 1 (baseline).

^b^T2: time point 2 (postintervention).

^c^EST: emotional image stress task phase.

^d^GM: guided meditation phase.

**Table 6:** Linear mixed effects model parameter estimates for heart rate variability among participants with posttraumatic stress disorder (PTSD) and participants without PTSD*a*.

Parameter			B (SE; 95% CI)	*t* test (*df*)	*P* value	Wald *Z*		*P* value	
**Fixed effects**							—^b^		—
	Intercept		*2.32 (0.12; 2.07 to 2.56)* ^c^	*18.77* (*67.74*)	<.001				
	**Treatment group**								
		Participants with PTSD	Reference	Reference	Reference				
		Participants without PTSD	*0.55* (*0.14; 0.26 to 0.84*)	*3.81* (*65.16*)	<.001				
	**Time period**								
		BL^d^	Reference	Reference	Reference				
		Postintervention	0.02 (0.06; –0.09 to 0.16)	0.411 (57.23)	.68				
	**Protocol condition**								
		BL	Reference	Reference	Reference				
		EST^e^ 1 (0-5 minutes)	*0.11* (*0.03; 0.05 to 0.16*)	*3.59* (*238.47*)	<*.001*				
		EST 2 (5-10 minutes)	0.05 (0.03; 0.00 to 0.11)	1.82 (257.16)	.07				
		GM^f^ 1 (0-5 minutes)	*0.14* (*0.03; 0.08 to 0.20*)	*4.67* (*257.86*)	<*.001*				
		GM 2 (5-10 minutes)	*0.08* (*0.03; 0.02 to 0.14*)	*2.64* (*286.98*)	.*009*				
Random effects (intercept)			*0.25* (*0.05; 0.17 to 0.36*)	—	—	*5.35*		<*.001*	
**Repeated measures**				—	—			<*.001*	
	AR^g^ 1 diagonal		*0.06* (*0.01; 0.05 to 0.07*)			*10.52*			
	AR 1 rho		–*0.51* (*0.06; –0.61 to –0.39*)			*9.45*			

^a^Akaike Information Criterion value of 179.93.

^b^Not available (no data).

^c^Italicized values indicate significant findings (*P*<.01; 2-tailed).

^d^BL: baseline (rest phase; 5 minutes).

^e^EST: emotional image stress task phase.

^f^GM: guided meditation phase.

^g^AR: autoregressive covariance structure.

#### Pupillometry Analysis

**Figure 3:**
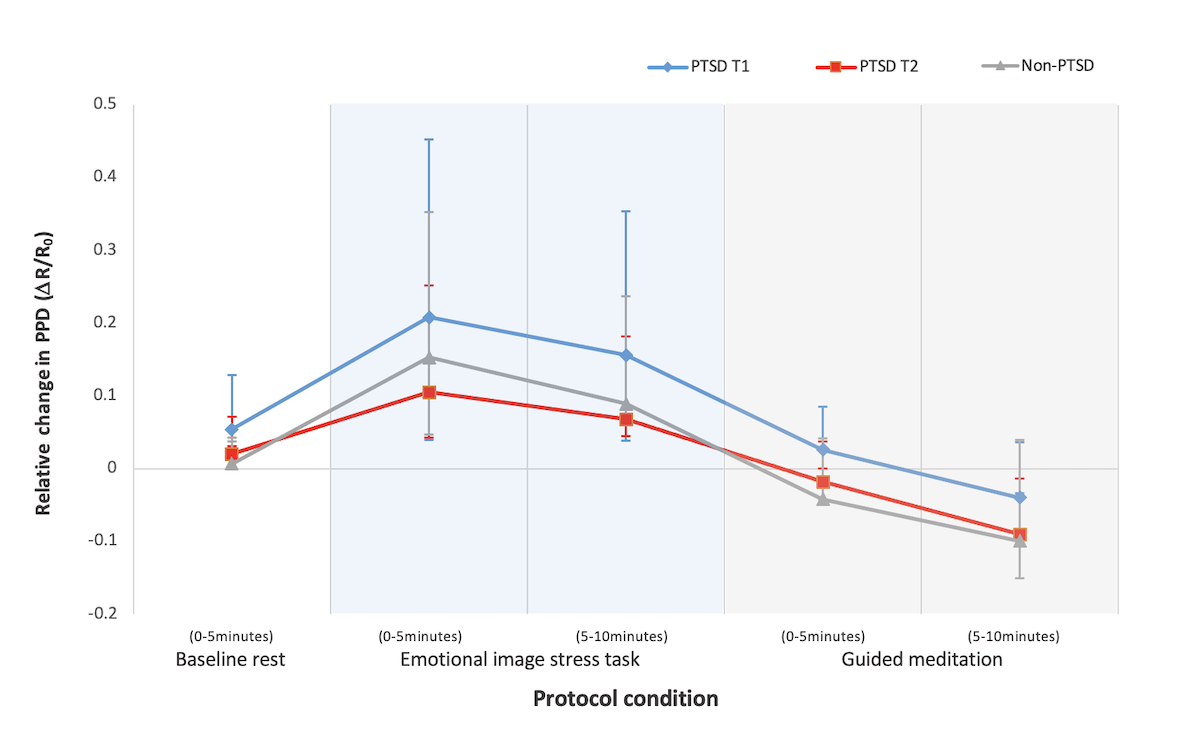
Mean change in relative PPD size across the 25-minute protocol. PPD is presented as relative pupil size and expressed as a ratio (0.10=10%) with 95% CI bars. PPD: peak pupil dilation; PTSD: posttraumatic stress disorder; T1: time point 1; T2: time point 2.

Figure 3 presents the mean fluctuations in the relative pupil diameter size across the entire 25-minute protocol. Changes in relative pupil size were significant across the protocol phases, with the EST phases (eg, 0-5 minutes, 5-10 minutes) showing the greatest increases in relative PPD, reflecting higher autonomic reactivity responses.

Descriptive statistics of the mean PPD outcome variables in participants with PTSD (18/64, 28%) and participants without PTSD (18/64, 28%) are reported in Table 7. LME analysis was conducted to determine where significant differences in PPD emerged.

As seen in Table 8, multilevel LME modeling revealed a significant main effect of protocol condition (*F*_4,148_=139.33; *P*<.001; ƞ^2^=0.79), time (*F*_1,54_=31.14; *P*<.001; ƞ^2^=0.37), and treatment group (*F*_1,35_=6.33; *P*=.02; ƞ^2^=0.15) on PPD. In terms of comparing participants with PTSD with participants without PTSD, pairwise comparisons revealed that participants without PTSD had a significantly lower overall PPD compared with participants with PTSD (B*=*−0.07; *t*_35_*=*–2.52; *P=*.02), reflecting reduced pupillary reactivity. Post hoc analyses to determine where significant differences emerged revealed that participants without PTSD had significantly lower PPD (mean 0.007, SD 0.06) during BL rest protocol condition compared with participants with PTSD (mean 0.05, SD 0.05) at T1 (*t*_34_=2.40; *P*=.02), indicating a large effect *(d=*0.78). Participants without PTSD also had significantly lower PPD during the first 5 minutes of GM (mean –0.04, SD 0.09) compared with participants with PTSD at T1 (mean 0.02, SD 0.07; *t*_34_=2.41; *P*=.02; *d*=0.74), which yielded a moderate to large effect size. At postintervention, no significant differences in PPD between participants with PTSD and participants without PTSD were found across any protocol condition, which supports our hypothesis and suggests that there was an intervention effect on reduced PPD reactivity among participants with PTSD.

For the main effect of time among participants with PTSD only, pairwise comparisons using Bonferroni type 1 correction revealed a significant reduction in overall PPD among participants with PTSD at postintervention compared with BL (Δmean=0.06, SE 0.01; *P*<.001), which equated to a large effect (*d*=0.90).

**Table 7:** Relative peak pupil dilation (PPD) across a 25-minute protocol among participants with posttraumatic stress disorder (PTSD) and participants without PTSD^a^.

PPD 25-minute protocol phases	Participants with PTSD, mean (SD)		Participants without PTSD, mean (SD)
	T1^b^ (n=18)	T2^c^ (n=17)	T1 (n=18)
Baseline (rest phase)	0.05 (0.05)	0.02 (0.07)	*0.00* (*0.06*)^d^
EST^e^ 1 (0-5 minutes)	0.21 (0.09)	0.10 (0.09)	0.15 (0.10)
EST 2 (5-10 minutes)	0.16 (0.10)	0.07 (0.10)	0.09 (0.13)
GM^f^ 1 (0-5 minutes)	0.03 (0.07)	−0.02 (0.08)	−*0.04* (*0.09*)^d^
GM 2 (5-10 minutes)	–0.04 (0.10)	−0.09 (0.08)	−0.10 (0.10)

^a^Peak pupil dilation values are presented as relative change from 0; a 0.10 change reflects a 10% increase in peak pupil dilation.

^b^T1: time point 1.

^c^T2: time point 2.

^d^Italicized values indicate significant difference (*P*<.05) between participants with and without posttraumatic stress disorder at time point 1.

^e^EST: emotional image stress task phase.

^f^GM: guided meditation phase.

**Table 8:** Linear mixed effects model parameter estimates for peak pupil dilation among participants with posttraumatic stress disorder (PTSD) and participants without PTSD^a^.

Parameter				Estimate (SE; 95% CI)		*t* test (*df*)			*P* value			Wald *Z*			*P* value	
**Fixed effects**													—^b^			—
	Intercept			*0.07* (*0.02*; *0.03 to 0.11*)^c^		*3.41* (*43.83*)			*.001*							
	**Treatment group**															
		Participants with PTSD	Reference		Reference			Reference								
		Participants without PTSD	–*0.07* (*0.03; 0.12 to –0.01*)		–2.52 (34.65)			.*02*								
	**Time period**															
		BL^d^	Reference		Reference			Reference								
		Postintervention	–*0.06* (*0.01; 0.08 to –0.04*)		–*5.58* (*53.72*)			<.*001*								
	**Protocol condition**															
		BL	Reference		Reference			Reference								
		EST^e^ 1	*0.13* (*0.01; 0.11 to 0.15*)		*11.37* (*162.12*)			<*.001*								
		EST 2	*0.08* (*0.01; 0.05 to 0.10*)		*6.80* (*144.07*)			<*.001*								
		GM^f^ 1	−*0.04* (*0.01; 0.06 to –0.02*)		–*3.58* (*145.74*)			<*.001*								
		GM 2	–*0.10* (*0.01; 0.12 to –0.08*)		–*9.13* (*153.38*)			<*.001*								
Random effects (intercept)				0.005 (0.00; 0.003 to 0.009)		—			—			*3.57*			<*.001*	
**Repeated measures**							—			—						
	AR^g^ 1 diagonal			0.004 (0.00; 0.003 to 0.005)								*10.25*			<.*001*	
	AR 1 rho			–0.21 (0.08; 0.36 to –0.04)								–*2.50*			.*01*	

^a^Akaike Information Criterion –577.92.

^b^Not available (no data).

^c^Italicized values indicate significant findings (*P*<.01; 2-tailed).

^d^BL: baseline rest phase.

^e^EST: emotional image stress task phase.

^f^GM: guided meditation phase.

^g^AR: autoregressive covariance structure.

### Exploratory Correlations Between PPD, HF-HRV, and PTSD Symptom Severity

Baseline resting PPD at T1 was significantly associated with CAPS-5 symptom severity score at postintervention (*r_p_=*0.54; *P=*.20) suggesting that a higher resting PPD (increased autonomic reactivity) is associated with a higher CAPS-5 symptom severity score at postintervention. HF-HRV at T1 (*P*=.21) or T2 (*P*=.69) was not significantly correlated with the CAPS-5 or PCL-5 PTSD symptom severity scores.

### Intervention Program Adherence

Overall, 94% (17/18) of participants had a 100% attendance rate on the weekly CBT calls, with one of the participants completing 88% (7/8) of the scheduled calls. In terms of weekly self-directed practice, the time spent in GM was highest (mean 38.89 min/week, SD 28.16 min/week), followed by yoga (mean 31.94 min/week, SD 28.24 min/week), and breathing techniques (mean 9.89 min/week, SD 11.87 min/week). Overall, participants spent 11.53 (SD 22.76) min/day in self-directed mindfulness (eg, meditation, yoga, breath awareness) practice. Combining all program intervention components (eg, CBT-MY), participants spent an average of 145 (SD 40.82) minutes (approximately 3 hours) per week engaging with the intervention, which is equivalent to 20 min/day. In total, 837 (SD 228.17) minutes of program engagement were recorded across the entire intervention equating to a total of 14-hours of program participation.

## Discussion

### Principal Findings

PTSD impedes daily function in multiple health domains [3,94]. Although significant findings support stand-alone CBT [95], mindfulness [41], and yoga treatment for PTSD [52], a paucity of research assesses integrated web-based approaches. This study investigated the psychometrics and objective measures of pupillometry and HF-HRV before and after an 8-week CBT-MY intervention. The results demonstrated significant self-reported improvements with large effect size reductions in PTSD (CAPS-5, *d*=1.60; PCL-5 *d*=1.27), depression (*d*=0.83), and anxiety (*d*=0.99) and increases in mindfulness (*d*=0.88). In objective measures, there were large effect size reductions for PPD (Δmean=–0.06, SE 0.01; *P*=.001), which were theoretically discrepant with the nonsignificant effects for HF-HRV (*P*=.87).

We found a large (*d*=1.60), clinically significant (≥10 points) reduction in CAPS-5 total symptom severity that exceeded the meta-analytic effect sizes referenced for psychotherapy for PTSD (*d*=1.43 [32]), web-based trauma-focused CBT (*d*=1.04 [54]), and web-based PTSD psychotherapy (*d*=1.05 [96]). The observed improvement was larger than the pre-post PCL-5 improvements associated with therapist-guided web-based PTSD therapy (*d*=1.40) [97]. The results compare favorably to a study of mindfulness and yoga in an intensive 3-week program for veterans diagnosed with PTSD associated with a pre-post effect size of Cohen *d*=1.12 [98]. In our study, BL depression severity moderate (mean 25.78, SD 13.39) and anxiety was severe (mean 28.33, SD 8.88). Postintervention scores reflected minimal depression (mean 11.28, SD 10.65) and mild to moderate anxiety (mean 15.72, SD 7.61 [99]). These reductions in depression and anxiety found in our study were consistent with previous trials showing comorbidity changes after effective PTSD treatment [54,98,100].

Although notable symptom improvements were observed in our study, they were partial improvements. At postintervention, 33% (6/18) of participants (compared with 20/22, 91% at BL) met the CAPS-5 PTSD diagnostic criteria, reflecting a reduction of 57.6%. Only one participant (1/18, 6%) reported being symptom-free at postintervention, whereas the remaining 61% (11/18) of participants reported subsyndromal PTSD [101]. McFarlane et al [102] proposed that varied PTSD symptom trajectories (eg, subsyndromal or partial PTSD vs severe or full PTSD) impact care provision and suggested that a clinical staging treatment strategy may be helpful to determine where specific treatment modalities are most effective. Symptom reductions based on PTSD trajectory can inform future clinical efforts by differentiating the stages of recovery [102]. With improved staging of PTSD recovery, clinical efforts can be targeted toward the specified participants most likely to achieve specific improvements [102]. Thus, the symptom improvements resulting from our CBT-MY intervention suggests a particular utility for a certain PTSD trajectory, but not necessarily appropriate for the most severe PTSD symptom trajectory. As noted in our findings, participants who scored *severe to extreme* PTSD symptom presentation in *Criterion D: negative cognition and mood symptoms* (CAPS-5) were the least likely to have reduced PTSD symptoms (to a subsyndromal stage) and most likely to withdraw from the intervention. On the basis of the recommendations by McFarlane et al [102], CBT-MY appears best suited for the less severe stages of clinical PTSD progression.

Participants exhibited substantial increases in 3 mindfulness facets (FFMQ), supporting the initial hypotheses. Specifically, improvements in *nonjudging* (*d*=0.96), *nonreactivity* (*d*=0.83), and *acting with awareness* (*d*=0.64) suggest participant integration of foundational mindfulness–yoga concepts (eg, *impermanence* and the appraisal of distressing events) [103]. For instance, more acceptance of change may facilitate *neutral judgments* of transient phenomena (memories and feelings) and *lesser reactiv*ity to associated distress [103,104].

The lack of FFMQ *observing* and *describing* improvements may be explained by a plausible interaction with hyperarousal symptoms [105]. This view is supported by research suggesting that articulating and communicating emotions becomes easier when amygdala hyperactivity, a PTSD neurocorrelate, is reduced [105,106]. Combined with the nonsignificant HF-HRV finding, it is possible that improvements in FFMQ *observing* and *describing* occur at a later stage in the PTSD recovery process [13].

Another plausible explanation is the presence of severe dissociative symptoms may have limited participants’ capacities for mindful attention [107]. Virtually all participants (21/22, 95%) disclosed clinical symptoms of dissociation (eg, depersonalization). Trauma-related dissociation involves avoidance, numbing, and the compartmentalization of psychological functioning and is often expressed in victims of childhood abuse with repeated exposure [108]. In turn, survivors of childhood abuse often experience difficulties in labeling and downregulating emotions [108]. Mindfulness practices aim to counteract such dissociative processes by promoting experiential integration through nonjudgmental, present moment awareness [35,51,107-110].

### Psychophysiology Outcomes

We found large effect size reductions for PPD (*d*=0.90) in contrast with nonsignificant HF-HRV effects (*P*=.87). Postintervention PPD and PTSD symptom outcomes (CAPS 5 and PCL-5) were correlated (*P*=.05), suggesting associations between self-report and PPD indicative of improved autonomic balance. HF-HRV results were discrepant, apparently unaffected by the intervention, and uncorrelated with self-reported benefits.

Participants with PTSD, compared with participants without PTSD, had significantly lower (pre- and postintervention) HF-HRV across all protocol phases. The mean BL resting HR in participants without PTSD was significantly lower than that in participants with PTSD. This finding is congruent with prior research examining the impact of trauma exposure and PTSD on autonomic dysregulation via HR and HF-HRV [111,112] and the findings of reduced capacities to psychophysiologically modulate in response to environmental changes [113,114]. During the lab procedure, the participants without PTSD exhibited significantly higher HF-HRV during stress onset (0-5 minutes) compared with that at rest, suggesting self-regulatory effectiveness and the mobilization of parasympathetic activity during negative image exposures [115]. In contrast, there were no significant changes in HF-HRV among participants with PTSD from rest to stress at BL or postintervention.

PTSD is often associated with comorbidities related to low and invariant HF-HRV [116-118]. It is possible that the chronicity of childhood trauma exposure (9.97 years duration) and severity of depression at BL had a blunting effect on HF-HRV during the protocol. Nonresponsive HF-HRV to a therapeutic intervention has been observed in patients with major depressive disorder [119,120]. Research examining the link between childhood emotional abuse and HF-HRV among women indicated that depressed women with histories of childhood emotional abuse exhibited significant HF-HRV decrements compared with depressed women without childhood abuse (*d*=0.90) and controls (*d*=0.87) [121]. These findings suggest that childhood emotional abuse is a strong predictor of impaired parasympathetic control.

Although PPD reductions across the 25-minute protocol were predicted, it is unclear why significant reductions were observed in PPD but not HF-HRV. An explanation involves mechanisms unique to ocular versus cardiac functioning. Theoretically, PPD reflects a more proximal and immediate form of threat reactivity than HF-HRV, as PPD responses appear to precede the autonomic reactions assessed by HF-HRV [21,113,122]. Although HF-HRV changes apparently reflect autonomic responses heavily influenced by cognitive–emotive responses, PPD changes reflect precognitive hypervigilance, hypothesized as prevalent in PTSD [21,113,122]. The varying foci of PPD and HF-HRV measurements in this study also substantially differed. PPD, representing autonomic function, was measured within the first 300 to 500 milliseconds of the onset of an image stimulus. This represents a significant duration difference from the recommended HF-HRV measurement of 5-minute epochs [92]. Not only does this indicate that PPD and HF-HRV measurement differences were, retrospectively, predictable as the observation period differed but also suggests that the physiological responses of participants differed significantly *during* image exposure. In the PPD response, participants were apparently less reactive to images at postintervention than at BL, whereas HF-HRV remained invariant and indicated sustained autonomic dysregulation at postintervention. Theoretically, the *ability* to *sustain* a less threatened response via HF-HRV is unlikely as participants are highly vulnerable to the more habit-based cognitive rumination and catastrophizing responses that *kick in* after an initially less disturbed image response [23]. Prior research has indicated an indirect relationship between HF-HRV and PPD, with low HF-HRV linked with perseverative cognition symptoms [123,124], which has been shown to be linked with greater pupil dilation to negative stimuli [125,126].

Few studies have explored the direct associations between HRV and the associated pupil response during rest, stress-induced, and meditation phases in clinical samples. Our findings are supported by a recent study by Macatee et al [127] that examined emotional processing indications of pupillary response and vagally mediated HRV in a nonclinical sample exposed to positive, negative, and neutral stimuli. HRV was unrelated to negative emotional processing and was not significantly associated with the pupillary indices of negative stimuli [127]. In contrast, low HRV predicted decreased pupil dilation to *positive* stimuli after a stress phase, reflecting altered *positive* emotional processing following stress induction [127]. As our study did not include positive emotional stimuli, a direct comparison was not possible. Future versions of our study should include positive emotional stimuli to better assess emotional processing in adults with PTSD via HRV and PPD and their associations.

A number of critical questions in our study merit further consideration. The first question is whether the selected negative emotional images were sufficiently evocative to elicit an autonomic stress response during the stress phase of the lab protocol. Our findings contrast with similar research conducted by Cascardi et al [128] that indicated that individuals who met PTSD diagnostic criteria showed large effect size differences (*d*=0.75) in pupil dilation to threatening stimuli compared with that of trauma-exposed controls without PTSD (*P*<.015). All the threatening images used by Cascardi et al [128] had an IAPS arousal rating of >6 [83]. The mean arousal rating of the images in our study was 5.51, and only 28% (17/60) of the selected images had an arousal rating of >6 [83,84]. Our preference for milder images, because of concern for the welfare of traumatized participants, may explain the nonsignificant PPD differences during the emotional image stress condition with the convenience sample of participants without PTSD.

However, another critical question is the extent to which the addition of positive stimuli might identify additional relationships between HRV and pupil response in participants with PTSD. In a recent cross-sectional study that examined pupil response to negative *and* positive images in individuals diagnosed with PTSD, McKinnon et al [21] found significantly larger pupil dilation in both threatening and positive (happy) images among individuals with PTSD than in controls exposed to trauma with no diagnosed PTSD (threatening, *d*=0.87; happy, *d*=0.73) and controls with no trauma exposure (threatening, *d=*0.85; happy, *d*=0.97). These findings suggest that autonomic function in PTSD is sensitive to *both* negative and positive image stimuli. Future studies of autonomic responses to a variety of emotional stimuli would advance the use of pupillometry as a PTSD biomarker. McKinnon et al [21] used a shorter image presentation (2000 ms) than our study (10 seconds), and each image was preceded and followed by a gray screen with a neutral fixation cross to allow the pupil to return to BL. Additional variations in used equipment and analysis procedures make precise comparisons difficult; however, the results from the McKinnon et al [21] study and our study findings lend support to further use of pupillometry in the assessment of emotional dysregulation and treatment of PTSD.

Our sample of participants reported spending an average of 12 min/day in self-directed mindfulness-based practice, which may have been sufficient to alter PPD-assessed precognitive autonomic function but insufficient to improve the autonomic regulation measured by HF-HRV. Prior controlled trials examining the effects of mindfulness on precognitive autonomic function have found significantly higher gray matter concentrations in the left hippocampal region of the brain in generally healthy meditators who spent 27 min/day in mindfulness [129] and changes in the connectivity of white matter fibers adjacent to the thalamus among long-term meditators (>15 years [130]). The thalamus relays immediate sensory information (as implicated in pupil dilation) and the hippocampal region modulates cortical arousal and emotional response [129,130]. Unfortunately, none of the referenced studies involved PPD measurements of autonomic function. Nonetheless, the reductions in PPD in our study, despite invariant HF-HRV, provide partial support for our hypothesized changes toward normative autonomic function after 8 weeks of the CBT-MY intervention. Given the known toxic effects of PTSD psychopathology on select brain regions (eg, decreased density of hippocampus), the lack of HF-HRV improvement is possibly explained by such effects [129].

Achieving states of safety, autonomic regulation via HF-HRV, and relaxation are challenging for individuals who are traumatized, and recovery rates vary in association with severity of PTSD symptoms, comorbidities, duration of trauma exposure, and time since last exposure [46,118,131,132]. Our sample of participants reported approximately double the number of direct trauma exposures than the national average [2] and disclosed childhood trauma that extended for a mean duration of 9.97 years, which has been associated with significant pathology across the life span and long-term deficits to health and overall functioning [118]. Nonetheless, the significant reductions in PPD found in this study appear to reflect positive intervention responses.

With positive changes in PPD, individuals with PTSD could experience environments as less dangerous and assess potential threats more normatively. Over time, such changes might be accompanied by positive responses in autonomic balance. Future randomized controlled trials (RCTs) examining longer intervention periods would help ascertain the ideal dosage of self-directed learning required to improve HRV. In this 8-week study, the effects appear sufficient to reduce hypervigilance, although not yet sufficient for improvements in HF-HRV.

### Strengths and Limitations

The strengths of this study include the rigorous clinical PTSD screening and assessment. The CAPS-5, a clinician-administered structured interview, is labor intensive but intended to be flexible and precise [63]. In addition, the PCL-5, a self-rated PTSD assessment, was used to precisely define and measure PTSD in conjunction with objective markers of autonomic function. The autonomic function of participants with PTSD and direct comparisons with a convenience cohort of relatively normative participants without PTSD were also conducted to assess psychophysiological differences.

There was a notable risk of experimenter bias with respect to PTSD assessments, as the CAPS-5 interview was conducted by a researcher who intervened with participants. However, there was a review process in which a trained second researcher with no relation to the study examined and verified all the scoring. As therapeutic alliances can significantly influence therapeutic outcomes, especially in the presence of childhood abuse [133], there was a possible confounding factor in the interview-based assessment. This issue was addressed through the additional use of the PCL-5, the self-rated counterpart to the CAPS-5, and the objective measures of autonomic function, especially PPD.

The attrition rate of the single-arm experimental trial was 18.2%, with 18% (4/22) of participants with PTSD not completing the postintervention follow-up measures, which limited a full understanding of the intervention effect. Of the 4 participants, 2 (50%) withdrew at week 2 and week 7, self-reporting child care obligations (week 2) and current trauma or stress exposure (week 7) as reasons, and the remaining 2 (50%) were lost to follow-up without providing any reason. On the basis of the CAPS-5 outcomes, the 4 participants who withdrew had significantly higher symptom severity for *Criterion D: negative cognition and mood symptoms*, suggesting that the intervention may be best implemented as an adjunctive treatment in combination with clinical psychological treatment addressing low mood or as a stand-alone program after acute mood symptoms and cognitions have improved. Other considerations such as time barriers and work or parenthood demands may be key factors to consider for future RCTs.

Although we controlled for multiple health factors in our research, our models did not account for the influence of pharmacotherapy on PPD and HF-HRV because of the small sample size and substantial heterogeneity in prescription type and dosage, as well as the lack of randomization. Evidence suggests that patients with a psychiatric diagnosis treated with tricyclic antidepressants demonstrate decreased HRV [134] as they contain anticholinergic compounds observed to reduce HRV. Tricyclic use could partially explain our nonsignificant HF-HRV results [134].

The results of this study must be interpreted with caution as they may not generalize to larger, more diverse samples. In addition to our small sample, the participants were mostly White, female, and highly educated. Nonetheless, these data provide a unique departure from the focus on war veterans in existing PTSD research. Other clear limitations include the lack of a randomly allocated waitlist control group, measurement blinding, and assessment of long-term changes and intervention effect maintenance.

### Conclusions

This study provides preliminary support for the effectiveness of web-based CBT-MY in the treatment of PTSD. The combination of CBT with mindfulness and yoga practice appears to summatively contribute to PTSD recovery. Future RCTs should examine whether the present findings extend to larger and more diverse samples and whether gains achieved are maintained over time. Undertreated populations with PTSD may be treated more effectively with web-based treatment and delivery methods that are more accessible, reducing the social and human costs of PTSD.

## Appendix

Multimedia Appendix 1 Weekly program modules in the 8-week cognitive behavioral therapy, mindfulness, and yoga program.

Multimedia Appendix 2 International Affective Picture System protocol images with emotional valence and arousal ratings.

## Abbreviations

ANS: autonomic nervous system
BAI: Beck Anxiety Inventory
BDI-2: Beck Depression Inventory-2
BL: baseline
BPI: Brief Pain Inventory
CAPS-5: Clinician-Administered Posttraumatic Stress Scale
CBT: cognitive behavioral therapy
CBT-MY: cognitive behavioral therapy, mindfulness, and yoga
DSM-5: Diagnostic and Statistical Manual of Mental Disorders, fifth edition
ECG: electrocardiography
EST: emotional image stress task
FFMQ: Five Facet Mindfulness Questionnaire
GM: guided meditation
HF-HRV: high-frequency heart rate variability
HR: heart rate
HRV: heart rate variability
IAPS: International Affective Picture System
ITT: intention-to-treat
LME: linear mixed effects
MM: mindfulness meditation
PCL-5: Posttraumatic Stress Disorder Checklist for the Diagnostic and Statistical Manual of Mental Disorders, fifth edition
PCS: Pain Catastrophizing Scale
PP: per-protocol
PPD: peak pupil dilation
PTSD: posttraumatic stress disorder
RCT: randomized controlled trial
T1: time point 1
T2: time point 2
