# Supplementary material for: A Web-Based Cognitive Behavioral Therapy, Mindfulness Meditation, and Yoga Intervention for Posttraumatic Stress Disorder: Single-Arm Experimental Clinical Trial
Source: JMIR Ment Health. 2022 Feb 28;9(2):e26479. doi: 10.2196/26479 (PMC8922150; doi:10.2196/26479)
Supplement: Multimedia Appendix 1 [file mental_v9i2e26479_app1.pdf]

Supplementary File:

Weekly program module themes in the 8-week CBT-MY program

**Module Topic**	**Major CBT-MY Themes**
1. Start Where You Are	- Present-moment awareness of bodily sensations, emotions and thoughts without judgement, discovering the fragmented self<br>- Mindfulness of avoidance and distractions<br>- Mindfulness of breath as an anchor to cultivate calm
2. The Ebb & Flow	- Challenging beliefs on what healing should look like<br>- Cultivating acceptance of the ebb and flow of healing<br>- Leaning into uncertainty, neutrality, and patience
3. Your Body Has Your Back	- Perspective shifting of bodily sensations and learning to sense the inner messages of the body<br>- Recognition of body cues; discernment of stress-response versus calm arousal<br>- Befriending the body with compassion
4. Emotional Triggers	- Addressing rumination, worry, and fear<br>- Understanding reactivity vs. responding<br>- Mindfulness of habitual negative thoughts, self-awareness, and knee-jerk reactions
5. Holding Space for Suffering	- Stress vs. trauma response in the body; dorsal vagal shut-down, dissociation, chronic hypervigilance<br>- Mindfulness for self-regulation and emotion regulation
6. The Second Arrow	- Uncovering cognitive distortions and negative self-talk<br>- Addressing shame, the Shadow Self and fragmented self<br>- Mindfulness as a tool for integrations of all ‘selves’
7. Towards Forgiveness	- Discovering mindfulness as a tool for self-compassion and compassion for others<br>- Understanding forgiveness through mindful breath to release and let go
8. Facing Forwards	- Cultivating inner peace, moving towards posttraumatic growth, resilience<br>- Rebuilding trust with inner sources of wisdom, mindfulness for communication, and authentic connection with self
